# Corytophanids Replaced the Pleurodont XY System with a New Pair of XY Chromosomes

**DOI:** 10.1093/gbe/evz196

**Published:** 2019-09-06

**Authors:** Armando Acosta, Gabriel Suárez-Varón, Luis A Rodríguez-Miranda, Andrés Lira-Noriega, Diana Aguilar-Gómez, Mariana Gutiérrez-Mariscal, Oswaldo Hernández-Gallegos, Fausto Méndez-de-la-Cruz, Diego Cortez

**Affiliations:** 1 Center for Genome Sciences, UNAM, Cuernavaca, Mexico; 2 Laboratorio de Herpetología, Facultad de Ciencias, Universidad Autónoma del Estado de México, Toluca, Mexico; 3 Biology Institute, UNAM, Mexico City, Mexico; 4 CONACYT Research Fellow, Red de Estudios Moleculares Avanzados, Instituto de Ecología, Xalapa, México; 5 Biotechnology Institute, UNAM, Cuernavaca, Mexico

**Keywords:** casque-headed lizards, *Basiliscus vittatus*, XY chromosomes, sex chromosome system turnovers, sex chromosome evolution

## Abstract

Almost all lizard families in the pleurodont clade share the same XY system. This system was meticulously studied in *Anolis carolinensis*, where it shows a highly degenerated Y chromosome and a male-specific X chromosome dosage compensation mechanism. Corytophanids (casque-headed lizards) have been proposed as the only family in the pleurodont clade to lack the XY system. In this study, we worked with extensive genomic and transcriptomic data from *Basiliscus vittatus*, a member of the *Corytophanidae* family that inhabits the tropical rainforests of Mexico. We confirmed that *B*. *vittatus* underwent a sex chromosome system turnover, which consisted in the loss of the pleurodont XY system and the gain of a new pair of XY chromosomes that are orthologous to chicken chromosome 17. We estimated the origin of the sex chromosome system to have occurred ∼63 Ma in the ancestor of corytophanids. Moreover, we identified 12 XY gametologues with particular attributes, such as functions related to the membrane and intracellular trafficking, very low expression levels, blood specificity, and incomplete dosage compensation in males.

## Introduction

Reptile species have undergone numerous sex determination systems turnovers, with lineages shifting more frequently from environmental-dependent sex determination (ESD) to genotypic sex determination (GSD) (Pennell et al. 2018). The second most-common transition in reptiles is transitions from GSD to ESD (Pennell et al. 2018). In general, very few cases of species shifting from one sex determination system to the same sex determination system, for instance, an XY system to a new XY system, have been documented (Bachtrog et al. 2014). Reptiles are an ideal taxon to test predictions on the mechanisms of sex determination because ESD and various types of GSD are present in closely related species of the same lineage and sex determination systems can be both of recent origin (<10-Myr old) or very old (>100-Myr old) (Bachtrog et al. 2014).

**Fig. 1.**
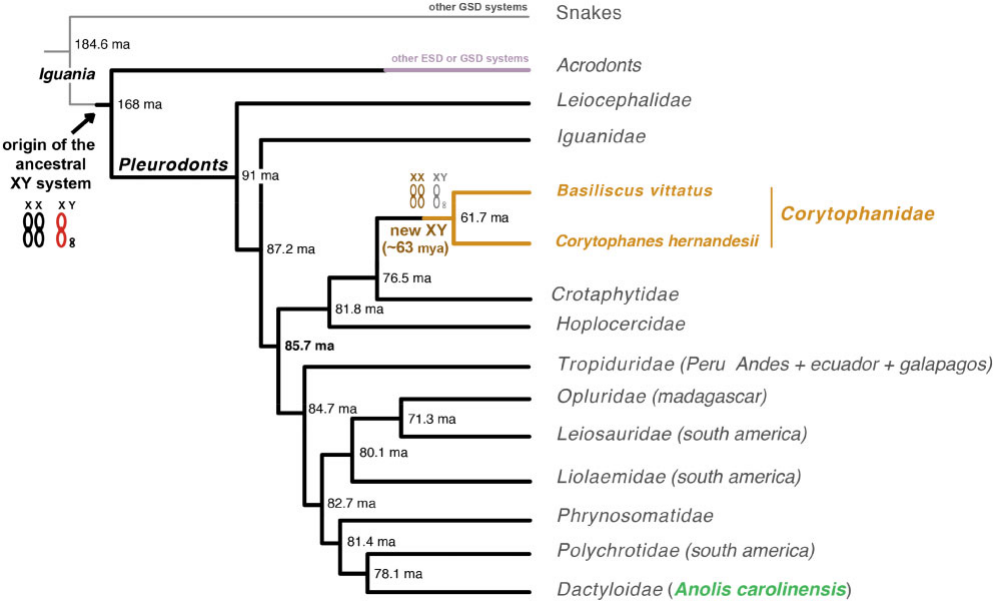
—Sex determination systems in the pleurodont clade. A tree representing the phylogenetic relationships between the families comprised in the pleurodont clade. The phylogenetic tree was based on Zheng and Wiens (2016). The estimate of *Basiliscus vittatus* and *Corytophanes hernandesii* divergence was taken from Taylor et al. (2017). Millions of years ago are denoted by “Ma.” Black and red XY chromosomes represent the pleurodont sex chromosome system, which originated in the ancestor of the infraorder *Iguania*. Most families in the pleurodont clade have conserved the same XY chromosomes; data based on Altmanova et al. (2018). This sex chromosome system has been well characterized in the green anole, *Anolis carolinensis* (highlighted in green), where it shows a highly degenerated Y chromosome and a male-specific X chromosome expression level upregulation; data based on Marin et al. (2017). The XY system in the *Corytophanidae* family originated 62.68 Ma (orange and gray XY chromosomes; results from this study). This corytophanid sex chromosome system is composed of heteromorphic sex chromosomes with a degenerated Y chromosome and an incomplete dosage compensation mechanism of the X chromosome in males.

For example, in the infraorder *Iguania*, the acrodont clade exhibits a great variety of ESD and GSD systems (Bachtrog et al. 2014), whereas all but one family of lizards in the pleurodont clade share the same sex chromosome system, which comprises a pair of heteromorphic XY chromosomes (Rovatsos, Pokorna, et al. 2014; Altmanova et al. 2018) (fig. 1). These XY chromosomes originated in the ancestor of the infraorder *Iguania*, 160–170 Ma (Marin et al. 2017) (fig. 1). This sex chromosome system has been studied with great detail in the green anole, *Anolis carolinensis* (*Dactyloidae* family) (Alfoldi et al. 2011; Gamble et al. 2014; Rovatsos, Altmanova, et al. 2014a, 2014b; Kichigin et al. 2016; Marin et al. 2017; Rupp et al. 2017). A previous work identified a highly degenerated Y chromosome in *A. carolinensis* that has conserved only a handful of genes (seven genes) (Marin et al. 2017). Additionally, it was also found that in order to maintain balanced gene expression levels in both sexes and counterweight the massive loss of genetic material of the Y chromosome, the X chromosome in *A. carolinensis* evolved a male-specific mechanism that triggers the hyper-acetylation of the lysine 16 on the histone 3, which in turn mediates the global upregulation of gene expression on the X chromosome (Marin et al. 2017).

In the pleurodont clade, only the species in the *Corytophanidae* family (casque-headed lizards) seem to lack the canonical XY system (Altmanova et al. 2018) (fig. 1). Two previous studies (Rovatsos, Pokorna, et al. 2014; Altmanova et al. 2018) examined four species of corytophanids (*Laemanctus longipes*, *Laemanctusserratus*, *Corytophanes hernandesii*, and *Basiliscus plumifrons*) and quantified the copy number of six genes that are X-linked in *A. carolinensis*. The researchers found that both males and females of corytophanids presented two copies of these genes. These results could indicate that the orthologous chromosome to the X chromosome in the closely related species *A. carolinensis* is an autosome in the corytophanids. However, the copy number of so few genes could also be influenced by X/Y gene conversion or translocations of genetic material between the X and Y chromosomes.

In this study, we used extensive genomic and transcriptomic data from the brown basilisk, *Basiliscus vittatus*, from a population that inhabits the tropical rainforests of Mexico, to confirm the loss of the pleurodont XY system. In addition, we identified and characterized a new XY system in corytophanids, which is based on chromosomes that are orthologous to the chicken chromosome 17. The corytophanid sex chromosomes originated in the ancestor of the family ∼62.68 Ma. We also explored the functions, sex determination candidates, expression levels, and evolution of the novel sex chromosome system.

## Materials and Methods

### Genome Data Generation, Assemblies, and Coverage Analyses

One adult male and one adult female individual of *B. vittatus* species were captured from a population that inhabits an open riverbed juxtapositioned to tropical rainforest habitat at the community of “La Selva del Marinero” in Veracruz, Mexico (170 m.a.s.l.; SEMARNAT Scientific Collector Permit 08-043). Sex was determined based on the size of the crests (larger in males), body size (males are bigger), and body coloration (wider yellow stripes in males). Later, sex was confirmed by the presence of ovaries or testis during the dissections. Both individuals were sacrificed using a lethal dose of pentobarbital; this study met legal regulations and institutional procedures for the investigation of the University of Mexico. Additionally, blood samples for *B. vittatus* (*n* = 6; three males and three females) and *C. hernandesii* (*n* = 6; three males and three females) individuals were obtained. We generated DNA-seq libraries for the male and female of *B*. *vittatus* from liver tissue using the Illumina TruSeq DNA protocol for short insert size (400–450 nt). The paired-end DNA-seq libraries were sequenced on Illumina HiSeq 2500 sequencers at the Macrogen facility in Korea (100-nt paired-end reads). We sequenced both genomes at approximately 6× of coverage based on the size of the *A.carolinensis* genome (1.7 Gb). The quality of the reads was verified using FastQC (http://www.bioinformatics.babraham.ac.uk/projects/fastqc; last accessed September 13, 2019) and the remaining adaptors were removed with Trimmomatic (Bolger et al. 2014). We then followed a methodology previously applied to the analysis of sex chromosomes in snakes (Vicoso et al. 2013). Specifically, the male and female raw genomic reads were assembled into scaffolds using SOAP de novo (v.2; default parameters) (Luo et al. 2012), and the resulting scaffolds were aligned against the six assembled chromosomes and the X-linked scaffolds from the *A. carolinensis* reference genome using BWA (bwa-mem) (Li et al. 2009). The selected scaffolds in the male and female *B. vittatus* genomes were required to align over 50% of their sequence length and above 80% of identity against the *A. carolinensis* genome. We ordered the *B. vittatus* scaffolds following the sequence of the chromosomes and X-linked scaffolds in *A. carolinensis*. We used bowtie2 (Langmead and Salzberg 2012) to align the raw DNA-seq reads from the male and female *B. vittatus* genomes onto the reconstructed chromosomes and X-linked scaffolds of *B. vittatus.* The aligned reads were sorted using SAMtools (Li et al. 2009) and the coverages for each chromosome (including the X chromosome) for males and females were calculated using BEDtools (Quinlan and Hall 2010). To compute the final data illustrated in the figures, we averaged the coverage using windows of 100,000 nucleotides for the 6 main chromosomes and windows of 10,000 nucleotides for the X chromosome. The novel X chromosome in *B. vittatus* was reconstructed using the sequence of the chicken chromosome 17, because the corresponding scaffolds in the *A. carolinensis* reference genome surpass the 100 fragments. We repeated the same procedure but changed the thresholds for the BWA analysis given that *B. vittatus* diverged before from chicken than from *A. carolinensis*. Selected scaffolds in the *B. vittatus* genomes were required to align over 40% of their sequence length and above 70% of identity against the chicken genome. Genomic data for *A. carolinensis* were taken from Marin et al. (2017).

### Transcriptome Data Generation and Assembly of Y-Linked Transcripts

We generated strand-specific RNA-seq libraries (using the Illumina TruSeq Stranded mRNA Library protocol) for a total of 14 samples obtained from blood (twice), brain, heart, liver, kidney, and gonads for both a male and a female of *B. vittatus*. Each library was sequenced on Illumina HiSeq 2500 platforms at the Macrogen facility in Korea (100 nucleotides, paired-end). In order to assemble Y-linked transcripts in *B. vittatus* we used a subtraction approach we applied previously in two studies in mammals/birds (Cortez et al. 2014) and *A. carolinensis* (Marin et al. 2017). We first assessed the quality of the reads from the 14 samples with FastQC (http://www.bioinformatics.babraham.ac.uk/projects/fastqc; last accessed September 13, 2019), trimmed the remaining adaptors with Trimmomatic (Bolger et al. 2014) and removed all reads with ambiguous nucleotides (N). Next, we collapsed all male RNA-seq reads from *B. vittatus* into one single file and aligned these reads onto the de novo reconstructed female genome from *B. vittatus* using Hisat2 (v2.0.2)(Kim et al. 2015); reads not mapping to this genome were selected. We then used all female RNA-seq data from *B. vittatus* to build an index of 35 bp k-mers; following a previous procedure (Akagi et al. 2014). We calculated the frequency of these 35 bp k-mers and removed those showing frequencies below ten; we did not consider rare k-mers as part of the overall signature of the female transcriptome. We used Bowtie2 (2.1.0) (Langmead and Salzberg 2012) to align the more abundant 35 bp k-mers to the male reads that did not align onto the *B. vittatus* female genome (with no mismatches and no indels allowed); we selected the male reads with no successful alignments. Finally, we assembled a male transcriptome with Trinity (v2.0.2, default k-mer of 25 bp) (Grabherr et al. 2011) based on the few selected read that passed all the filters. We obtained putative male-specific transcripts, which were aligned against the raw DNA-seq reads from the male and female *B. vittatus* using BlastN (Altschul et al. 1990). We searched for transcripts having 100% identity over 90% or more of their sequence length in the male genome and no significant alignments in the female genome. Twenty-three transcripts were only found in the male genome. To further reinforce our results, we decided to amplify a subset of six Y-linked genes using genomic PCRs and DNA from *B. vittatus* blood samples (*n* = 6; three males and three females). The primers used were: *COL1A1* (autosomal/control) forward: TTT CGT GCA GGG TGG GTT CTT T, reverse: TCT GAA CTG GTG CAG CTT CAC A. Whereas Y-linked genes were: *CAMSAP1*, forward: AGT CTC AGT CTG CAC CAG TGA AAG, reverse: TGA TTT CTG AGC CCA GGC AGT T. *GOLGA2*, forward: AGG CTG TCA GTC TCA CTC AGT AAG, reverse: CCC CAT ATT CCC AGG TTC TGT CA. *EHMT1*, forward: TCT CCC AGG GTT ACG AAC GGA T, reverse: TGT CTA CGG AAT TGA CGC AGG GAA. *RAB14*, forward: GTG CCT TTG GCT GCT TCG TTT T reverse: ATG TGC TAG GCC TGC AAT GAG T. *HSPA5*, forward: TGT TTT GGA AGG CAC GCA GCT A, reverse: TCG TCA TCG TCA GCA AAC ACA C. *ZBTB34*, forward: TCC TGC CAA ACA GTG ACC AGA T, reverse: AGC ACC TCA TGG CTG GTT GT. *NIBANY/FAM129BY*, forward: AGC CGG GTC ATC GCC TCC TG, reverse: CTC TCG TCC AGG TGC GCT GAG. *CACNA1BY*, forward: GCA CAG TTG GCA ATG ATC AAG T, reverse: GGT CAA CAA ACA TCC CTC TGG CA. Gene with unknown function, forward: CCG CAA CAG CCC TAT CAG CCA, reverse: ACC TGT TGC AGT ATC CTT CAG CT. *MEGF9Y*, forward: GCC CTC CTC GAC ATG ACA TCC C, reverse: AGC CAG TGA TGA TGA ACC TAC AG. *AKIY*, forward: AGC CAT TCT ACC CCG CTC CAA, reverse: TCG AGA GCC CAA AAC CAC GTC T. These primers were designed to specifically amplify Y sequences; accordingly, PCR reactions with female DNA did not show any amplification. The same PCR procedure was repeated using purified DNA from *C. hernandesii*. 0.2–0.5 ml of blood was extracted from the caudal vein with a 1 ml needle from 3 males and 3 females of *B. vittatus* and *C. hernandesii* (*n* = 6 for each species). Blood was immediately mixed with a buffer containing heparin and conserved a 4 °C. The wound on the animal skin was disinfected and the animals were released 24 h later at the same location where they were captured based on the recorded GPS coordinates. Male/female blood DNA was extracted following the manufacturer’s instructions using the Kit Blood DNA Isolation Mini de Norgen Biotek (cat. 46300). PCRs were performed with the Phusion Flash High-Fidelity PCR Master Mix (Thermo Fisher Scientific; cat. F548S) 30 cycles at 98 °C for 2 s, 66 °C for 5 s, and 72 °C for 10 s. PCR reactions were run on agarose gels (2%). Initially, we did not know whether *B. vittatus* presented XY or ZW chromosomes. So, in parallel, we ran the methodology starting with female RNA-seq data to determine the presence of potential W-linked sequences. All potential W-linked sequences were found in both the male and female genomes and represented false-positive transcripts.

### Search for *A. carolinensis* Y-Linked Genes

We applied a previously established genomic approach to search for Y genes using the orthologous genes found in other species (in this case, *A. carolinensis*) on the basis of high-throughput genomic sequencing data (Cortez et al. 2014; Marin et al. 2017). We figured that using orthologous sequences of Y and X gametologues, one could identify the orthologous genes from the genomic raw sequencing data in targeted species. Thereby, we used the known Y-linked genes from *A. carolinensis* and searched for their best matching reads (best identity) in the *B. vittatus* male and female raw DNA-seq reads using BlastN (Altschul et al. 1990). We then assembled the best target sequence (higher identity) and compared the resulting sequences against the sequences obtained with the same methodology but using the X gametologues from *A. carolinensis*. In both cases, using either the Y or the X gametologues, we reconstructed the orthologous gene to the *A. carolinensis* X-linked gene. Thus, we ruled out the presence of any *A. carolinensis* Y-linked genes that might have escaped the pleurodont Y chromosome by retrotransposition, transposition, or chromosomal fusion.

### Assignment of Y Gene Names

To establish Y gene identity, we searched NCBI GenBank (http://www.ncbi.nlm.nih.gov/genbank; last accessed September 13, 2019) for the closest homologs using BlastN and BlastX (Altschul et al. 1990). Transcripts without any significant match and without clear open reading frame predictions were considered to be noncoding. We note that the RNA-seq-based Y transcript reconstructions are expected to usually yield the most frequent isoform for a given Y gene. Functions of Y-linked genes were obtained from genecards (http://www.genecards.org; last accessed September 13, 2019).

### Synonymous Substitution Analyses

To estimate the approximate age at which XY gametologues halted homologous recombination, pairwise alignments of coding sequences of XY gametologues were obtained using PRANK (Loytynoja and Goldman 2005) based on encoded amino acid sequences. *d*_S_ values were then calculated using codeml (pairwise option) as implemented in the PAML package (Yang 1997). Six XY gametologues presented *d*_S_ values above 0.2 and five XY gametologues presented values below 0.2 that could represent older and younger gametologues, respectively. An older (stratum 1) and a younger (stratum 2) strata were later confirmed by a PCR approach using genomic DNA from *C. hernandesii* (see main text).

To assess the age at which the novel XY system was originated, we followed a previous procedure (Cortez et al. 2014). Briefly, we used the codeml free-ratio model, as implemented in the PAML package (Yang 1997). For each pair of the six XY gametologues in stratum 1 (see above; table 1) we aligned using PRANK (Loytynoja and Goldman 2005) the coding sequences of XY genes in *B. vittatus* and the coding sequences from one-to-one orthologous genes in *A. carolinensis*, chicken, the soft-shell Chinese turtle, opossum, mouse, human, and *Xenopus;* orthologous coding sequences were downloaded from the Ensembl database (https://www.ensembl.org/; last accessed September 13, 2019; v.92). We concatenated the individual gene alignments and selected random positions from the concatenation to generate a new alignment with the original length; we repeated this operation 100 times (100 bootstrap rounds). For each round, we calculated the synonymous substitutions for all branches of the species tree using codeml (implemented in PAML; Yang 1997). The average branch lengths from the 100 bootstraps were calibrated to produce an ultrametric, time-calibrated tree, using the “chronos” library (from the “ape” package in R). We then retrieved the branch lengths just before and after the split of the XY gametologues and the time since corytophanids diverged from *A. carolinensis* (outgroup species). We calculated the age of the sex chromosomes based on the individual branch lengths that together are equivalent to the time that has passed since the divergence between corytophanids and *A. carolinensis*. 95% confidence intervals for the sex chromosome origin estimate were obtained from the estimates of the 100 individual replicates. The divergence time between corytophanids and *A. carolinensis* was retrieved from the recent time-calibrated phylogeny for *Squamata* reported in Zheng and Wiens (2016) and the weighted-median of the divergence estimate reported at TimeTree (http://www.timetree.org/; last accessed September 13, 2019).

**Table 1:** Protein-Coding Y-Linked Genes Identified in *Basiliscus vittatus*

	Gene	Type	*d*_S_	*Y* gene in *Corytophanes hernandesii*	Strata	*Anolis* Chromosome	Chicken Chromosome	Human Chromosome	Function
1	*NIBANY/FAM129BY*	Protein-coding	0.42	Yes	1	GL343556	17	9	Family with sequence similarity 129 member B
2	*CAMSAP1Y*	Protein-coding	0.36	Yes	1	GL343625	17	9	Calmodulin regulated spectrin associated protein 1
3	Unknown	Protein-coding	0.27	Yes	1	NA	NA	NA	Hypothetical zinc finger
4	*EHMT1Y*	Protein-coding	0.26	Yes	1	GL343556	17	9	Euchromatic histone lysine methyltransferase 1
5	*CACNA1BY*	protein-coding	0.24	Yes	1	GL343556	17	9	Calcium voltage-gated channel subunit alpha1 B
6	*AKIY*	Protein-coding	0.22	No	2	AAWZ02036994	17	9	Adenylate kinase isoenzyme 1
7	*MEGF9Y*	Protein-coding	0.17	No	2	GL343398	17	9	Multiple EGF like domains 9
8	*RAB14Y*	Protein-coding	0.11	No	2	AAWZ02036218	17	9	Member RAS oncogene family
9	*HSPA5Y*	Protein-coding	0.088	No	2	AAWZ02037731	17	9	Heat shock protein family A (Hsp70) member 5
10	*GOLGA2Y*	protein-coding	0.083	No	2	GL343763	17	9	Golgin A2
11	*ZBTB34Y*	Protein-coding	0.069	No	2	GL343502	17	9	Zinc finger and BTB domain containing 34
12	*CCDC183Y*	Protein-coding	NA	No	2	NA	NA	9	Coiled-coil domain containing 183

### RNA-Seq Read Mapping and Expression Analyses

We concatenated the RNA-seq data from the 12 samples from *B. vittatus* and reconstructed a full transcriptome using Trinity (v2.0.2, default k-mer of 25 bp) (Grabherr et al. 2011). Then, RNA-seq reads were mapped to the reconstructed transcriptome using Kallisto (Bray et al. 2016). We downloaded from the Ensembl database (https://www.ensembl.org/; last accessed September 13, 2019; v.92) the transcriptomes of *A. carolinensis* and chicken. RNA-seq reads reported in a previous study (Marin et al. 2017) were mapped to the Ensembl transcriptomes of *A. carolinensis* and chicken using Kallisto. Gene expression estimates (transcripts per million or TPM) were calculated by Kallisto (100 bootstraps). For each gene in the *B. vittatus*, *A. carolinensis*, and chicken transcriptomes we selected the transcript with the maximum expression level. Normalization across samples was performed using a previous median scaling procedure (Brawand et al. 2011) that uses one-to-one orthologous genes expressed in all samples to obtain correcting indexes. Comparisons of expression levels between genes on current/ancestral and male/female chromosomes were carried out as previously described (Julien et al. 2012; Cortez et al. 2014; Marin et al. 2017). Specifically, to infer ancestral expression levels we exploiting the fact that current sex chromosomes are derived from ancestral autosomes and, therefore, have autosomal counterparts in species with nonhomologous sex chromosomes, which are informative concerning proto-sex chromosome expression patterns. We calculated ancestral sex chromosome expression levels as median expression levels of autosomal one-to-one orthologues of X genes in outgroup species with different sex chromosomes systems: *A. carolinensis* and chicken. Ancestral inferred expression output values were calculated per one gene copy/allele, that is, the obtained values were divided by 2. The tissue-specificity index (TSI) for a given gene was calculated as the expression level (TPM) in the tissue with the highest expression level divided by the sum of expressions values in all tissues (Julien et al. 2012).

### Statistical Analyses

All statistical analyses were performed using the R package, standard libraries. Data were plotted using the R package, “ggplot2” library (https://ggplot2.tidyverse.org/; last accessed September 13, 2019).

## Results

### Corytophanids Lost the XY System Present in All Other Families of Pleurodonts

**Fig. 2.**
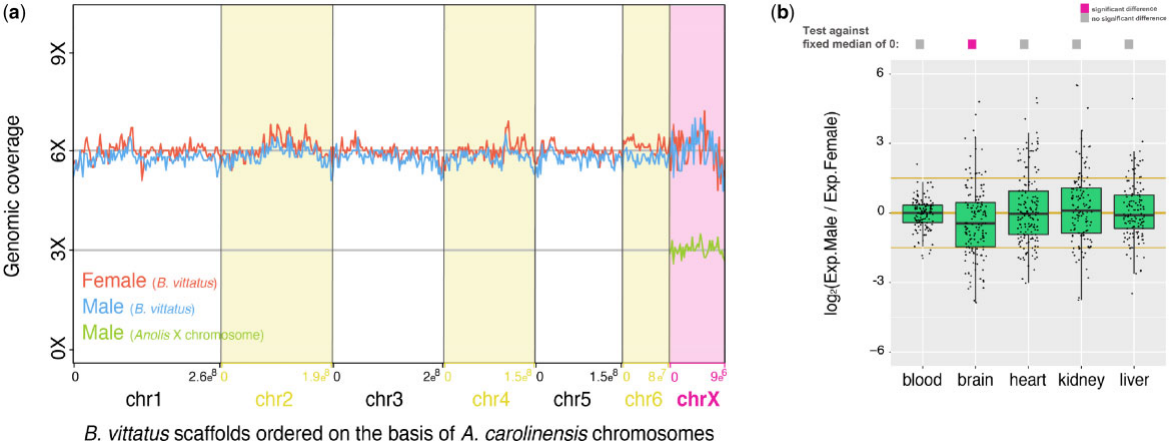
—Analysis of the pleurodont X chromosome in *Basiliscus vittatus*. (*a*) Coverage analyses using either male (blue line) or female (red line) genomic reads for the six main autosomes and the pleurodont X chromosome. Autosomes and the pleurodont X chromosome in *B. vittatus* were assembled on the basis of *Anolis carolinensis* reference genome. The expected coverage of an X chromosome in males with heteromorphic XY chromosomes is exemplified by the genomic coverage of the X chromosome in *A. carolinensis* (green line); genomic data for *A. carolinensis* were taken from Marin et al. (2017). (*b*) Boxplots representing the male/female expression ratio of genes located on the pleurodont X chromosome (*n* = 218 genes) in somatic tissues. Significant differences (Mann–Whitney *U* test): Benjamin–Hochberg-corrected *P *<* *0.05 of temperatures against a distribution with fixed median of 0 (i.e., similar expression levels of X genes in males and females). Gray filled squares denote nonsignificant differences between male/female ratios of X genes against a distribution with fixed median of 0, whereas pink filled squares denote significant differences. Error bars, maximum and minimum values, excluding outliers.

We sequenced (6× of coverage) and assembled into scaffolds the genomes of one male and one female individuals of *B. vittatus*. The scaffolds were identified and ordered based on their identity to the *A. carolinensis* reference genome (see Materials and Methods), assuming general conservation in gene synteny between the two species. Analyses of the six main autosomes (chromosomes 1–6) resulted in the same genomic read coverage for males and females (fig. 2*a*, blue and red lines). Importantly, we also observed the same genomic read coverage in both sexes for the orthologous sequences to the X chromosome in *A. carolinensis* (fig. 2*a*, blue and red lines), hence this chromosome is present in two copies in the male and female genomes of *B. vittatus* and it is indistinguishable from any other autosome. In contrast, in species that harbor heteromorphic XY chromosomes, such as *A. carolinensis*, the X chromosome shows half of the genomic read coverage in males (one X chromosome present; fig. 2*a*, green line) compared with females (two X chromosomes present). Scaffolds corresponding to the pleurodont X chromosome in *B. vittatus* are described in supplementary table 1, Supplementary Material online.

Moreover, the old X chromosome in *B. vittatus* exhibited the same expression levels in most somatic tissues in males and in females (except for brain that shows female-bias expression; Benjamini–Hochberg-corrected Mann–Whitney *U* test against a reference value of “0,” *P *<* *0.05; fig. 2*b*), indicating that the old X chromosome shows balanced expression levels between sexes and that the male-specific X upregulation mechanism described in *A. carolinensis* (Marin et al. 2017) is probably lost. Lastly, we searched the male and female genomes of *B. vittatus* for *A. carolinensis* Y-linked genes that could have either escaped the Y chromosome via retrotransposition or were translocated to a different chromosome. We could not find traces of any known Y-linked genes from *A. carolinensis*, meaning the pleurodont Y chromosome was completely lost from *B. vittatus* lineage.

### Corytophanids Acquired a New Pair of XY Chromosomes

**Fig. 3.**
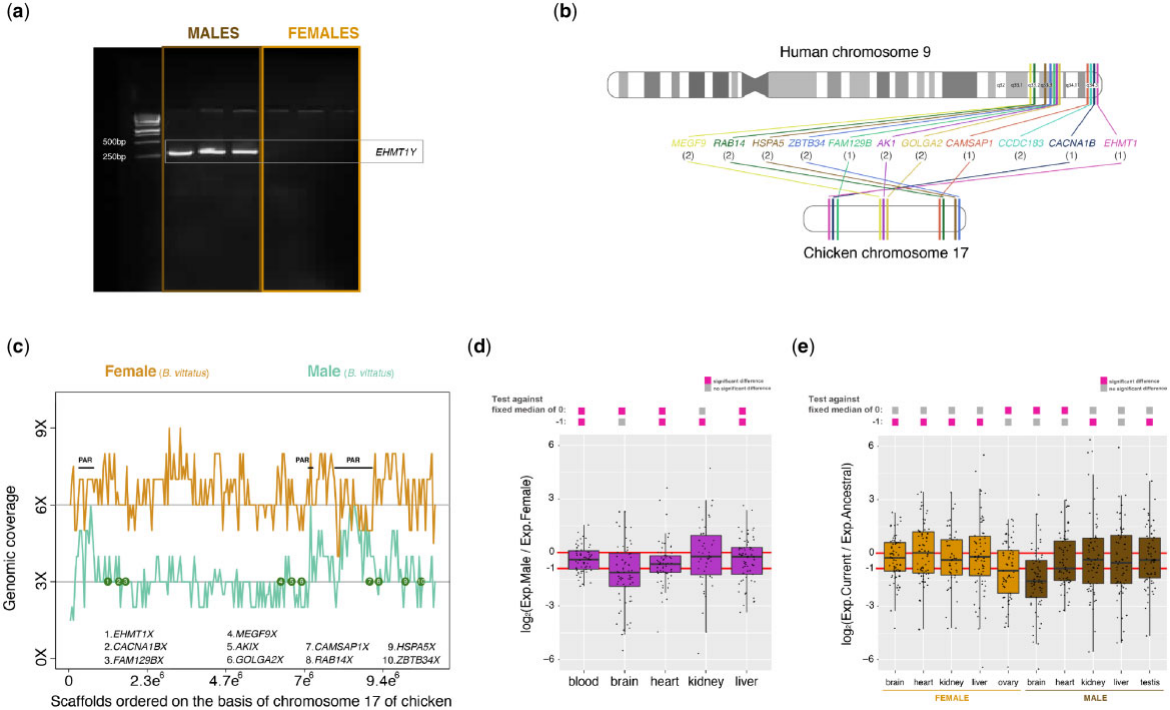
—Analysis of the novel XY chromosomes. (*a*) Agarose gel showing the PCR products of the *EHMT1Y* and *COL1A1* genes. The control gene, *COL1A1*, was amplified in both males and females, whereas the exemplified Y-specific gene, *EHMT1Y*, was amplified only in males. (*b*) Diagram depicting the chromosomal positions in human and in the chicken of the orthologous genes to the XY gametologues in *Basiliscus vittatus*. The numbers correspond to the strata assignments (see Table 1 and main text). (*c*) Coverage results for the novel X chromosome in *B. vittatus* using either male (green line) or female (orange line) genomic reads. The X chromosome in *B. vittatus* was assembled on the basis of the chicken chromosome 17. Green circles denote the approximate location of the X gametologues; numbers correspond to the genes shown in the figure. (*d*) Boxplots representing the male/female expression ratios of genes located on the novel X chromosome (*n* = 67 genes) in somatic tissues. Significant differences (Mann–Whitney *U* test): Benjamin–Hochberg-corrected *P *<* *0.05 of temperatures against distributions with fixed medians of 0 (i.e., similar expression levels of X genes in males and females) and −1 (i.e., half the expression levels of X genes in males compared with females. (*e*) Boxplots representing the current/ancestral expression ratio for genes located on the novel X chromosome (*n* = 67 genes) for female tissues (light brown) and male tissues (dark brown). Significant differences (Mann–Whitney *U* test): Benjamin–Hochberg-corrected *P *<* *0.05 of temperatures against distributions with fixed medians of 0 (i.e., current and ancestral expression levels are similar) and −1 (i.e., current expression levels are half of the ancestral expression levels). (*d*–*e*) Gray filled squares denote nonsignificant differences between male/female ratios of X genes and a distribution with fixed medians (0 or −1), whereas pink filled squares denote significant differences. Depending on the dispersion of the data, a sample could be either not significant or significantly different from the two fixed medians. Error bars, maximum and minimum values, excluding outliers.

We applied a subtraction approach to the male and female transcriptomic data of *B. vittatus*. We used RNA-seq data from brain, heart, liver, kidney, and gonads of one male and one female *B. vittatus*. Briefly, we searched for RNA-seq reads that were only present in males or females. We then assembled sex-specific transcriptomes and aligned the resulting transcripts to the male and female genomic reads to discard those genes with sex-specific expression that are not truly located on sex chromosomes (see Materials and Methods). Out of 3,185 transcripts with male-biased expression, we found that 23 transcripts were present in the male genome of *B. vittatus* and absent from the female genome of *B. vittatus*. Of these 23 male-specific transcripts, 12 code for proteins (table 1), whereas 11 appear to be long noncoding RNAs (no hits in public databases and with no obvious open reading frames) or transposable elements. We validated six protein-coding Y genes employing genomic PCRs (see Materials and Methods), obtaining PCR amplifications only when male DNA was used (fig. 3*a*).

We noted that the protein-coding Y genes from *B. vittatus* were not orthologous to genes located on the X chromosome of *A. carolinensis*. Instead, they were orthologous to genes located on various scaffolds in this species with no chromosomal assignment (table 1). Despite the long evolutionary distance, we searched the Y-linked genes of *B. vittatus* in the reference genome of chicken. We found that all of them are located on chromosome 17, which is also orthologous to the final segment of the *q* arm on the human chromosome 9 (*q*33–*q*34; fig. 3*b*;table 1).

Detailed examination of the scaffolds in *B. vittatus* that are orthologous to the chromosome 17 in chicken showed that the genomic read coverage in males is half (3×) of the genomic read coverage found in females (6×; fig. 3*c*). Thus, *B. vittatus* shows a fully differentiated pair of XY chromosomes. Importantly, the X gametologues in *B. vittatus* are located on the nonrecombinant region (i.e., the male-specific region) of the sex chromosome (fig. 3*c*). Of the 287 protein-coding genes annotated on the chromosome 17 of chicken (https://www.ensembl.org/Gallus_gallus/Location/Chromosome?r=17; last accessed September 13, 2019), which could serve as a proxy of the ancestral gene content, we recovered 95% of the genes on the X chromosome in *B. vittatus* but only 13 (4.5%) genes on the Y chromosome in *B. vittatus*. Based on these results, we inferred that most of the Y content has been lost. Note that the coverage between males and females is similar (6× of coverage) in three small segments of the chromosome sequence (fig. 3*c*; PAR labels). These segments likely represent the pseudoautosomal region, which appears divided into three segments probably due to chromosomal rearrangements that occurred following the divergence of chicken and the corytophanids.

We found that the expression levels of the corytophanid X chromosome are biased toward females in blood, brain, heart, and liver tissues (fig. 3*d*). Male/female expression level ratios of X-linked genes are different from a distribution with fixed median of 0 (i.e., similar expression levels of X genes in males and females), and are also different from a distribution with fixed median of −1 (i.e., half the expression levels of X genes in males compared with females). Therefore, the male/female expression level ratios for the X chromosome are placed at an intermediate state. This suggests that the X chromosome in males is neither fully compensated nor shows the absence of dosage compensation. Expression level analyses indicate that most X-linked genes are partially compensated. Data from kidney showed data with larger variance and were not significant against the reference value of zero.

We decided to compare the current expression levels of the genes on the corytophanid X chromosome against the estimated ancestral expression. To infer ancestral expression levels, we exploited the fact that the current sex chromosomes are derived from ancestral autosomes and therefore have autosomal counterparts in species with nonhomologous sex chromosomes, which are informative concerning proto-sex chromosome expression patterns. We estimated ancestral expression levels using one-to-one orthologues of X genes in *A. carolinensis* and chicken. We found that the X chromosome in female tissues maintained the ancestral levels (except for ovary that shows lower current expression levels). In males, the pattern exhibited by the X chromosome seems more complex: brain, heart, and liver show reduced current expression levels, whereas kidney and testis have higher current expression levels.

### Functions and Gene Expression Patterns of XY Gametologues in Corytophanids

The gene set found on the Y chromosome of *B. vittatus* is different from those usually described on the Y chromosomes of mammals (Hughes et al. 2010; Bellott et al. 2014, 2017; Cortez et al. 2014; Janecka et al. 2018), the W chromosome of birds (Zhou et al. 2014), or the Y chromosome of *A. carolinensis* (Marin et al. 2017), where many of the few remaining Y and W-linked genes have regulatory functions. In *B. vittatus*, however, only two genes show clear regulatory functions: *EHMT1Y* (transcription regulation) and *ZBTB34Y* (transcription repression). The majority of the Y-linked genes in *B. vittatus* code for proteins with functions associated with the membranes and intracellular trafficking (table 1), such as microtubule-organization (*CAMSAP1Y*), membrane protein (*MEGF9Y*), intracellular membrane trafficking (*RAB14Y*), Golgi-specific protein (*GOLGA2Y*), voltage-dependent calcium channel (*CACNA1BY*), and a member of the axoneme (*CCDC183Y*). Additionally, we found a regulator of phosphorylation (*FAM129BY*), a chaperone (*HSPA5Y*), and an intracellular adenine ratio sensor (*AK1Y*). Associated functions were retrieved from the GeneCards database (https://www.genecards.org/; last accessed September 13, 2019).

**Fig. 4.**
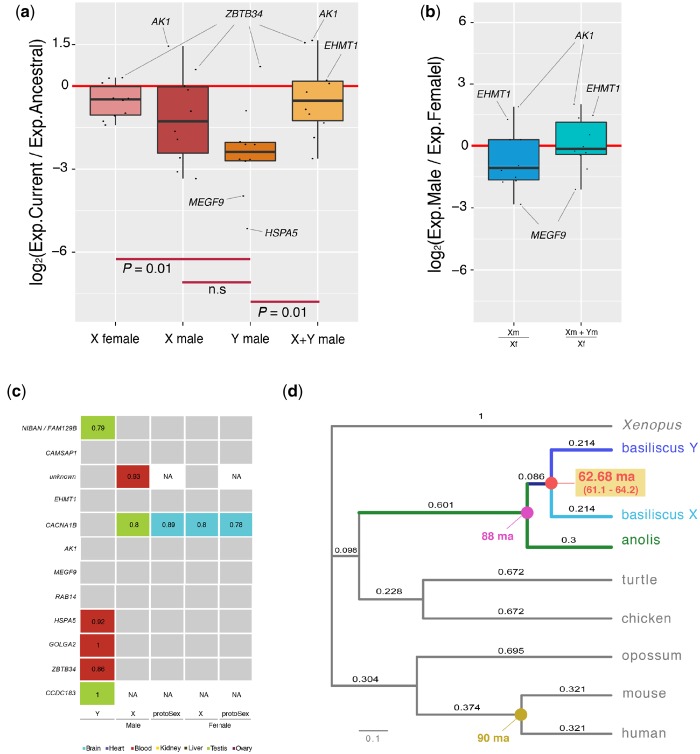
—Analyses of XY gametologues. (*a*) Boxplots representing the current/ancestral expression ratio of X gametologues in females, X gametologues in males, Y gametologues in males, and the added expression of X and Y gametologues in males (*n* = 10 genes). Significant differences (Mann–Whitney *U* test). Genes with the highest and lowest expression ratios are indicated. (*b*) Boxplots representing the expression balance of XY gametologues between the two sexes, that is, the X gametologues expression levels in males compared with those in females, as well as the added expression levels of X and Y gametologues in males compared with the expression levels of the X gametologues in females (*n* = 10 genes). Genes with the highest and lowest expression ratios are indicated. (*c*) Tissue-specificity index (TSI) of XY gametologues and proto-sex genes (tissue-specificity of orthologous genes in the chicken and the green anole) in males and females. Genes with a tissue-specificity index below 0.7 are colored in gray. Genes with a tissue-specificity index above 0.7 are filled using the colors shown at the bottom of the figure; TSI values are indicated. (*d*) *d*_S_ tree built using the concatenated coding nucleotide sequences of stratum 1 genes and orthologous sequences in other tetrapod species. The age of the sex chromosome system in corytophanids was obtained by comparing specific branch lengths and the age at specific nodes (see Materials and Methods). Age estimates for specific nodes are based on Zheng and Wiens (2016). Millions of years ago are denoted by “Ma.”

We also found that X gametologues in both females and males, which are not located in the pseudoautosomal region (fig. 3*c*), are lowly expressed compared with the ancestral expression levels (minus 1-fold change; fig. 4*a*). Moreover, with the exception of *ZBTB34Y*, Y-linked genes are very lowly expressed (minus 2.5-fold change; fig. 4*a*). However, the added expression of Y and X gametologues in males is similar to the expression of X gametologues in females (fig. 4*b*), meaning selection has probably acted to maintain a balanced expression level despite the low expression levels of the Y-linked genes.

Interestingly, *EHMT1Y-X*, a gene that codes for a protein capable of modifying the epigenetic landscape of the genome (https://www.genecards.org/; last accessed September 13, 2019), retained the ancestral expression levels in males when the expression levels of both gametologues were combined (fig. 4*a*–*b*). Finally, in corytophanids, following Y expression decay, very few genes changed their tissue-specificity. Most genes were expressed in all tissues at similar levels (fig. 4*c*). One gene gained testis specificity (*NIBAN*/*FAM129BY;*fig. 4*c*), one gene appears to be unique to the Y chromosome and shows testis specificity (*CCDC183Y;*fig. 4*c*), three genes gained blood specificity (*GOLGA2Y*, *HSPA5Y*, and *ZBTB34Y;*fig. 4*c*), and one gene gained kidney specificity (*MYO6Y;*fig. 4*c*).

### Origin of the New XY System in Corytophanids

To define the presence of potential strata on the sex chromosomes of *B. vittatus*, we first calculated *d*_S_ values between XY gametologues. Six genes showed *d*_S_ values greater than 0.2 and could represent older gametologues (table 1). Five genes showed *d*_S_ values smaller than 0.2 and could represent younger gametologues (table 1). Finally, one gene, *CCDC183Y* could not be analyzed because the X gene was absent from our database.

The XY chromosomes in *B. vittatus* could be unique of the species or could be present in other species of corytophanids. Previous studies (Rovatsos, Pokorna, et al. 2014; Altmanova et al. 2018), however, indicate that several species of corytophanids most likely replaced the pleurodont sex chromosomes by another sex determination system. However, multiple sex determination systems could have evolved in the family. To confirm the presence of the XY system in other corytophanids we sought to amplify by means of genomic PCR the 12 Y-linked protein-coding genes from *B. vittatus* in DNA samples from *C. hernandesii*; this species belongs to the second major clade in the *Corytophanidae* family (fig. 1). The five Y genes with the largest *d*_S_ values, *NIBANY/FAM129BY*, *CAMSAP1Y*, the gene with unknown function, *EHMT1Y*, and *CACNA1BY* showed PCR amplification specifically in males (table 1; supplementary fig. 1, Supplementary Material online, for an example), indicating that *C. hernandesii* shares the XY system with *B. vittatus*. According to the fossil record, *B. vittatus* and *C. hernandesii* diverged ∼61.7 Ma (fig. 1) (Taylor et al. 2017). Finally, we classified the XY gametologues in two strata: five genes in stratum 1, which halted recombination before the split of *B. vittatus* and *C. hernandesii* (>61.7 Ma) and seven genes in stratum 2 that are probably exclusive of the *B. vittatus* lineage (<61.7 Ma).

Since the split between the *Corytophanidae* and *Crotaphytidae* (sister group) (Zheng and Wiens 2016) families occurred ∼76.5 Ma (fig. 1) (Zheng and Wiens 2016), then the sex chromosome system turnover in corytophanids occurred at some point over a period of ∼14.8 Myr. In order to obtain a more precise estimate for the origin of the corytophanid XY system, we performed analyses based on *d*_S_ trees. We built the trees using the nucleotide sequences of the XY gametologues in *B. vittatus* and orthologous sequences from *A. carolinensis*, chicken, mammals, and *Xenopus*. We compared the branch lengths of the ultrametric, time-calibrated tree, X and Y sequences against the branch length just before their divergence. We calibrated the values based on the divergence time between *A. carolinensis* and *B. vittatus* using the most recent time-calibrated phylogeny of squamates (85.7 Ma; Zheng and Wiens 2016; figs. 1 and 4*d*; see Materials and Methods). From the concatenated data of stratum 1 gametologues we estimated that the origin of the corytophanid XY system occurred ∼61.05 Ma (95% confident intervals: 59.55–63.1 Ma, values derived from 100 bootstrap rounds; fig. 4*d*).

The age of the sex chromosomes is based on specific branch lengths in the *d*_S_ trees and the most recent time-calibrated phylogeny for squamata (Zheng and Wiens 2016). However, alternative phylogenies may affect the inferences we draw from evolutionary processes (Title and Rabosky 2017). We, therefore, repeated the analysis using the time estimate retrieved from the TimeTree database (http://www.timetree.org/; last accessed September 13, 2019), which reports the weighted-average divergence estimate from multiple scientific studies. The *A. carolinensis*/*B. vittatus* split reported in Time Tree is of 88 Ma. We estimated the origin of the XY system to have taken place around 62.68 (61.1–64.2 Ma; 95% confident intervals).

## Discussion

In this study, we confirmed that corytophanids lost the pleurodont XY system that, otherwise, has been preserved in all other families of the pleurodont clade (31 species from 12 families of pleurodonts have been examined so far) (Gamble et al. 2014; Rovatsos, Pokorna, et al. 2014; Altmanova et al. 2018) for 160–170 Myr. Moreover, we showed that corytophanids acquired a new pair of XY chromosomes. The pleurodont Y chromosome was completely lost from corytophanids after the emergence of a new sex-determining gene able to control the signaling cascade that triggers the development of gonads. The pleurodont Y-linked genes were most likely lost because they conserved functional redundancy with the X gametologues and did not evolve significant male-specific functions. This hypothesis could be tested in pleurodonts with the ancestral XY system. We also found that the new sex chromosome system in corytophanids has several attributes that are unique compared with other sex chromosomes that have been studied in amniotes. For instance, in mammals, the X gametologues are among the X-linked genes with the highest expression levels (Cortez et al. 2014), and Y-linked genes show regulatory functions that could be beneficial to males, for example, in spermatogenesis. In contrast, Y-linked genes in *B. vittatus* are very lowly expressed, show membrane or intracellular trafficking-related functions and particular tissue-specificity gains, such as blood- and kidney-specific expression. The membrane and transport-related functions of most Y-linked genes are also at odds with the master regulatory role Y chromosomes are supposed to play, a process that is generally driven by genes coding for transcription factors or epigenetic regulators. After screening the Y-linked catalog of *B. vittatus*, the gene *EHMT1Y*, a histone lysine methyltransferase, or the gene *ZBTB34Y*, a transcription repressor, could be promising candidates for sex determination. The *ZBTB34Y* gene, however, is preferentially expressed in blood, which does not fit well with activity during sex determination. The *EHMT1Y* gene shows broad conserved expression across many somatic tissues, which is also not compatible with a role as a sex-determining regulator. More experiments would be needed to identify the sex-determining gene on either the Y or X chromosomes in corytophanids.

In theory, the X chromosome in females should show twice the expression level compared with males when the Y chromosome has degenerated and there is no dosage compensation mechanism. We propose that males of corytophanids exhibit incomplete dosage compensation, that is, most genes are partially compensated (i.e., relatively lower expression levels in males compared with females). A similar model was proposed for the sex chromosomes in platypus and chicken (Julien et al. 2012).

Gametologues are subjected to specific selection forces because males have maintained an X and Y active alleles that could potentially produce a dosage conflict. In humans, for instance, because one X chromosome is inactivated in females, the X gametologues tend to escape the inactivation process (Bellott et al. 2014) to preserve balanced expression levels between males and females. In *B. vittatus*, XY gametologues show balanced expression levels in both sexes, despite their low expression levels. It has been suggested that Y- and W-linked genes were probably selected due to their haploinsufficiency (i.e., the quantity of proteins produced by one allele is insufficient to carry out the biological function) (Bellott et al. 2014; Cortez et al. 2014). Our results suggest that this hypothesis fits well with the data found in *B. vittatus*.

We classified the XY gametologues in two strata. Stratum 1 is composed of five genes that halted recombination before the split of *B. vittatus* and *C. hernandesii* (>61.7 Ma). Using the synonymous substitution rates of these genes we estimated that the “old-XY system -> new-XY system” transition occurred ∼62.68 Ma in the ancestor of the *Corytophanidae* family. Note that this estimate could change as new fossils of casqued-head lizards are found (Taylor et al. 2017).

We were unable to identify which environmental or genetic factors could have triggered, ∼63 Ma, the sex chromosome system transition in corytophanids. New methods are therefore required to reconstruct the species’ ancestral geographical ranges, which could help associate specific sex chromosome system transitions with past environmental fluctuations.

## Supplementary Material

Supplementary data are available at *Genome Biology and Evolution* online. 

## Supplementary Material

evz196_Supplementary_Data

## Notes

**Data deposition:** This project has been deposited at the NCBI database (http://www.ncbi. nlm.nih.gov/) under the accession BioProject PRJNA525415. Y-linked sequences from *B. vittatus* have been deposited at the figshare platform: https://figshare.com/s/9387c059b18c14cf42e4. The reconstructed transcriptome of *B. vittatus* has been deposited at the figshare platform: https://figshare.com/s/c45f79ac89e17d648619. The reconstructed genomes of *B. vittatus* have been deposited at the figshare platform: https://figshare.com/s/b04db76fb38021ee27ed. Expression data used in the study is listed in the Supplementary table 2, Supplementary Material online.
